# Low methylthioadenosine phosphorylase expression is associated with worse survival in patients with acute myeloid leukaemia

**DOI:** 10.1002/ctm2.70015

**Published:** 2024-09-11

**Authors:** Yiyu Xiao, Qianqian Peng, Advaith Maya Sanjeev Kumar, Houda Alachkar

**Affiliations:** ^1^ Department of Pharmacology and Pharmaceutical Sciences USC Alfred E. Mann School of Pharmacy and Pharmaceutical Sciences University of Southern California Los Angeles California USA; ^2^ Department of Computer Science University of Southern California Los Angeles California USA; ^3^ Department of Clinical Pharmacy USC Alfred E. Mann School of Pharmacy and Pharmaceutical Sciences University of Southern California Los Angeles California USA; ^4^ USC Norris Comprehensive Cancer Center University of Southern California Los Angeles California USA

Dear Editor,

Deletions of methylthioadenosine phosphorylase (*MTAP*) are frequent in several malignancies and lead to 5′‐deoxy‐5′‐methylthioadenosine (MTA) accumulation, competing with S‐adenosylmethionine (SAM) for binding to Protein Arginine Methyltransferase 5 (PRMT5) and enhancing tumour sensitivity to PRMT5 inhibitors.^1^, ^2^ Although MTAP enzyme deficiency has been documented in acute myeloid leukaemia (AML), deletions of the MTAP gene have not been identified in this haematological malignancy. Here we evaluated MTAP downregulation in AML datasets (TCGA and OHSU)^3^, ^4^, ^5^, ^6^, ^7^ and its associations with clinical and molecular characteristics and patient's clinical outcome.

When comparing the *MTAP* expression between AML bone marrow (BM) (*n* = 542) and healthy BM (*n* = 73), different *MTAP* probes showed different results (MILE dataset,^8^ Figure 1A–F). However, when comparing the GTEx and TCGA datasets on UCSC Xena,^9^ we found higher *MTAP* expression in AML blood (*n* = 173) than normal blood samples (*n* = 337) (median‐log2: 4.040 vs. −0.199, *p *= 6.596e^−197^, Figure 1G). Thirteen transcripts of MTAP were differentially expressed (Figure 1H). While only one deep deletion case was identified in AML in the TCGA dataset, using Z‐score < −1 to define low expressor *MTAP*, we found 10.40% and 16.84% of cases have low *MTAP* expression in the TCGA and OHSU, respectively (Figure 1I).

**FIGURE 1:**
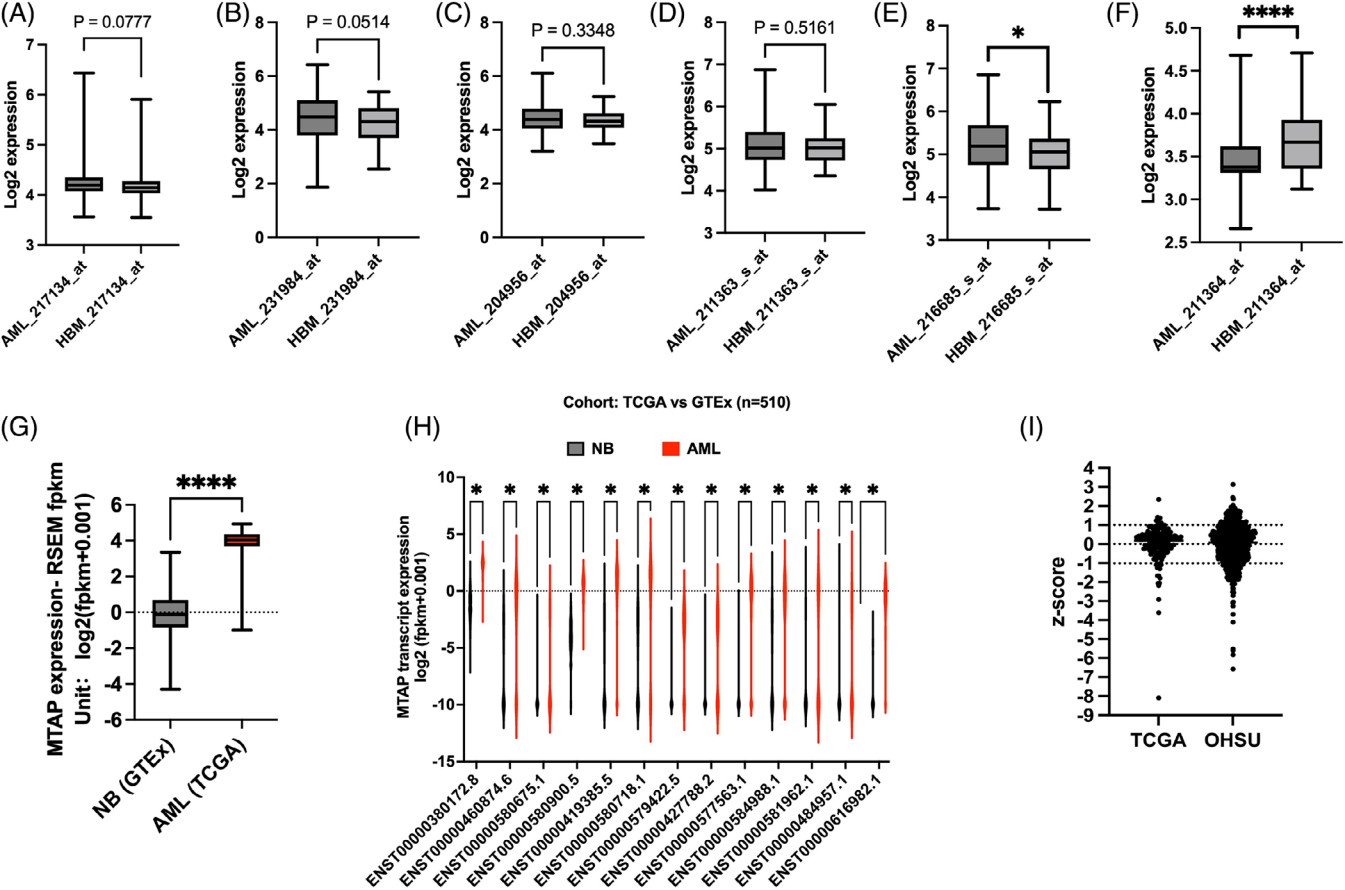
Methylthioadenosine phosphorylase (*MTAP*) expression in acute myeloid leukaemia (AML) and normal specimens and the distribution of *MTAP* z‐score in the OHSU and TCGA datasets. (A–F) *MTAP* expression in healthy bone marrow (HBM: *n* = 73) and AML bone marrow (*n* = 542) in the MILE dataset. Six different probes were used to measure MTAP. (G) *MTAP* expression in normal whole blood samples (NB: *n* = 337) and white blood cells in AML patients’ peripheral blood (AML: *n* = 173) was compared by Welch's t‐test. (H) Expression of 13 *MTAP* transcripts in normal whole blood samples (NB: *n* = 337) and white blood cells in AML patients’ peripheral blood (AML: *n *= 173). Protein coding transcripts are marked by arrows (ENST00000580900.5, ENST00000460874.6, ENST00000427788.2 and ENST00000577563.1). (I) Z‐score of *MTAP* of all available samples with RNA‐seq data in TCGA and OHSU. (**** *p* < .0001; *** *p* < .001; ** *p* < .01; * *p* < .05; except for H: * q < 0.05).

To investigate whether low expression of *MTAP* is associated with specific baseline clinical features in patients with AML, we compared the frequency of *MTAP* low expression according to diagnosis age, sex, BM blast percentage, white blood cell count, peripheral blasts percentage, cytogenetic risk and molecular risk (Tables S1 and S2). In the TCGA dataset, we found *MTAP* low expression to be more frequent in older patients (diagnosis age ≥ 65 years) than in younger patients (diagnosis age < 65 years) (18.87% vs. 6.67%, *p *= .027). *MTAP* expression levels were found to be lower in patients with AML M3 (*n* = 16) than AML M2 (*n* = 38) (*p* = .030, Figure 2A).

**FIGURE 2:**
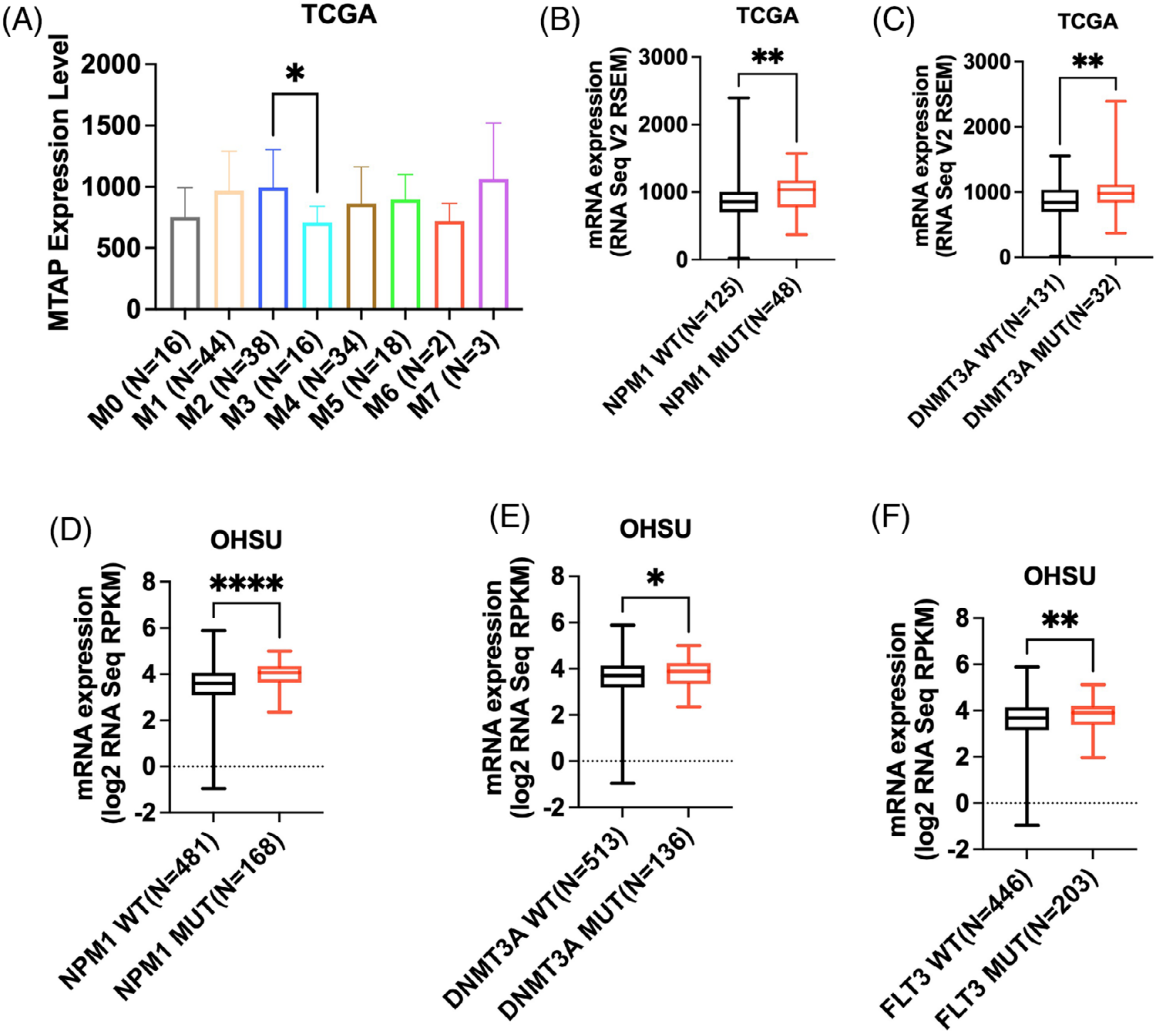
(A) Expression of methylthioadenosine phosphorylase (*MTAP*) regarding FAB (M0–M7) in M0 (*n* = 16), M1 (*n* = 44), M2 (*n* = 38), M3 (*n* = 16), M4 (*n* = 34), M5 (*n* = 18), M6 (*n* = 2) and M7 (*n* = 3). Comparison of *MTAP* expression in TCGA dataset between (B) 48 patients with NPM1 mutations (MUT) and 125 patients with NPM1 wild type (WT); (C) 32 patients with DNMT3A mutations and 131 patients with DNMT3A wild type. Comparison of *MTAP* expression in OHSU dataset between (D) 168 patients with NPM1 mutations and 481 patients with NPM1 wild type; (E) 136 patients with DNMT3A mutations and 513 patients with DNMT3A wild type; (F) 203 patients with FLT3 mutations and 446 patients with FLT3 wild type. Dunn's test was used for calculating adjusted P values. (**** *p* < .0001; *** *p* < .001; ** *p* < .01; * *p* < .05).

We also assessed the association between *MTAP* low expression and AML molecular characteristics in terms of the presence of certain AML mutations (Tables S3 and S4). We compared the frequencies of *FLT3*, *DNMT3A*, *NPM1*, *IDH2*, *IDH1* and *TP53* mutations between low and unaltered/high *MTAP* patients. In OHSU, patients with MTAP low expression have a lower frequency of FLT3 mutations (17.3% vs. 32.1%, *p* = .036) and NPM1 mutation (3.8% vs. 30.6%, *p* < .001) compared with unaltered/high MTAP group. *MTAP* was expressed at significantly higher levels in patients with *NPM1* mutation (median‐log2, TCGA: 1037 vs. 857.5, *p *= .0018, adjusted‐*p* = .0112; OHSU: 4.058 vs. 3.606, *p *< .001, adjusted‐*p* < .001), *DNMT3A* mutation (median‐log2, TCGA: 980.3 vs. 844.0, *p* = .0094, adjusted‐*p* = .0569; OHSU: 3.887 vs. 3.702, *p* = .048, adjusted‐*p* = .288), *FLT3* mutation (median‐log2, OHSU: 3.905 vs. 3.670, *p* = .007, adjusted‐*p* = .044), compared with patients carrying wild type genes (Figure 2B–F).

Survival analyses showed that the overall survival (OS) of *MTAP*‐low patients was significantly shorter than that of unaltered/high *MTAP* patients (*MTAP*‐low patients vs. *MTAP*‐ unaltered/high patients; median‐OS [months]: TCGA: 7.5 vs. 20.5, *p *= .014; OHSU: 10.16 vs. 17.79, *p *= .02, Figure 3A,B). Additionally, the disease‐free survival (DFS) of MTAP‐low patients was significantly lower than *MTAP*‐unaltered/high patients in the TCGA dataset (*MTAP*‐low patients (*n* = 18) vs. *MTAP*‐unaltered/high patients (*n* = 153); median‐DFS (months): 8.200 vs. 17.00, *p *= .017, Figure 3C). The OHSU dataset does not contain DFS data. We also conducted survival analyses after the exclusion of patients with AML M3, and patients with no available FAB data, due to the favourable prognosis of all‐trans retinoic acid therapy. *MTAP*‐low patients still have worse outcomes compared with *MTAP*‐unaltered/high patients (TCGA: *MTAP*‐low patients (*n* = 16) vs. *MTAP*‐unaltered/high patients (*n* = 139); median OS (months): 7.200 vs. 17.40, *p *= 0.004, OHSU: *MTAP*‐low patients (*n* = 50) vs. *MTAP*‐unaltered/high patients (*n* = 349); 10.16 vs. 15.52, *p *= 0.035, Figure 3D,E). Multivariable analysis (cox‐regression model) showed that *MTAP* low expression is significantly associated with OS (TCGA: *p *= 0.026; OHSU: *p *= 0.031) when adjusted by age, *DNMT3A‐*mutation, *TP53‐*mutation, *FLT3‐*mutation (Table 1).

**FIGURE 3:**
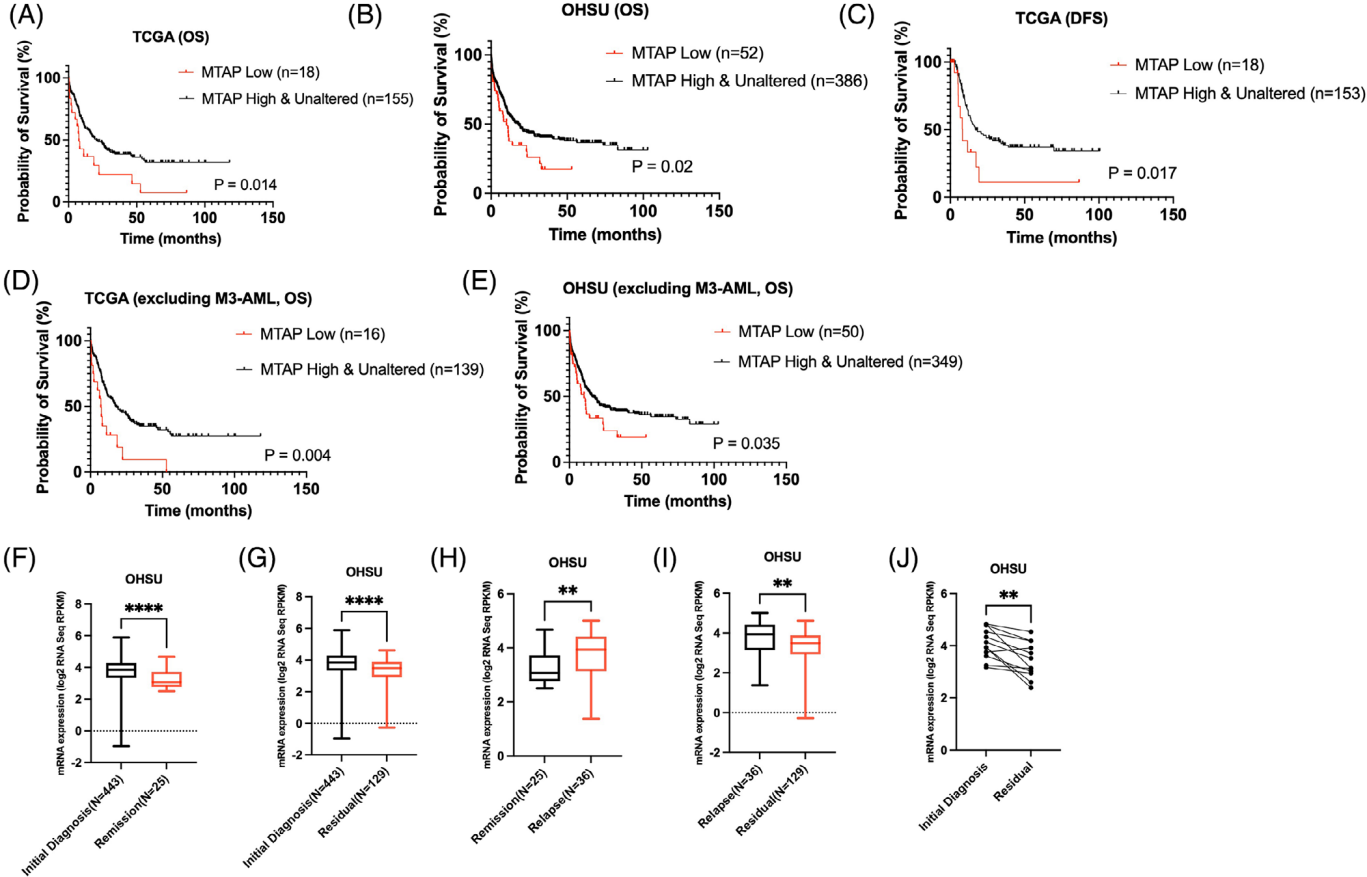
Association between *MTAP* expression and AML clinical outcome. (A and B) Overall survival of methylthioadenosine phosphorylase (*MTAP*) low and *MTAP* unaltered/high patients in TCGA dataset and OHSU dataset. (C) Disease‐free survival of *MTAP* low and *MTAP* unaltered/high patients (*n* = 171) in TCGA dataset. (D and E) Overall survival of *MTAP* low and *MTAP* unaltered/high patients excluding patients with M3‐AML in the TCGA and OHSU dataset. (F–I) Expression of *MTAP* of 443 initial diagnosis samples, 25 remission samples, 36 relapse samples and 129 residual samples in the OHSU dataset. (J) Paired analysis of 12 samples between initial diagnosis samples and residual samples. (**** *p *< .0001; *** *p *< .001; ** *p *< .01; * *p *< .05).

**TABLE 1:** Multivariable analysis.

Variable	|Z|	*p*
TCGA		
Low MTAP expression	2.222	.0263
Diagnosis age ≥65 years	4.568	<.0001
DNMT3A mutant type	1.535	.1248
TP53 mutant type	2.057	.0396
Cytogenetic & molecular risk: Intermediate	2.026	.0428
Cytogenetic & molecular risk: Poor	3.693	.0002
FLT3 mutant type	2.604	.0092
OHSU		
Low MTAP expression	2.155	.0312
Diagnosis age ≥65 years	8.342	<.0001
DNMT3A wild type	0.06665	.9469
TP53 mutant type	6.024	<.0001
FLT3 wild type	1.537	.1243

When comparing *MTAP* expression at different disease statuses in the OHSU dataset (initial diagnosis, remission, residual and relapse), we found *MTAP* expression levels were significantly higher at diagnosis (*n* = 443) than at remission (*n* = 25, median‐log2: 3.855 vs. 3.073, *p *< .001, Figure 3F) or at residual disease (*n* = 129, median‐log2: 3.855 vs. 3.491, *p* < .001, Figure 3G). *MTAP* expression was significantly higher at relapse (*n* = 36) than at remission (*n* = 25) (median‐log2: 3.938 vs 3.073, *p *= .003, Figure 3H) and at residual disease (*n* = 129) (median‐log2: 3.938 vs. 3.491, *p *= .002, Figure 3I). Consistently, *MTAP* expression levels were significantly higher in initial diagnosis compared with that in patients with residual disease when comparing samples from the same patients (*n* = 12, *p* = .001, Figure 3J).

Studies using synthetic lethal screens have shown that *MTAP*‐deleted cells exhibit higher sensitivity to downregulation of PRMT5. *MTAP*‐deleted cancer cells are particularly vulnerable to further inhibition of PRMT5 by the MTA‐cooperative PRMT5 inhibitor MRTX1719.^2^ This therapeutic approach selectively inhibits the PRMT5‐MTA complex in *MTAP*‐deficient cells.

MTA is generated through polyamine synthesis in which arginine is metabolized into ornithine and then into polyamines. Polyamine metabolism plays a key role in leukaemia stem cell survival and presents a potential therapeutic target in AML.^10^ It is plausible that MTA accumulation plays an important metabolic vulnerability in AML cells, and thus MTA levels should be evaluated in patients with AML. Therapeutic strategies that are proven effective in *MTAP*‐deleted cancers such as PRMT5 inhibitors and MTA‐cooperative PRMT5 inhibitors should be investigated in the context of low MTA AML.

Altogether, our study reveals the association between low *MTAP* expression and shorter overall survival and the absence of *NPM1* mutation in patients with AML. Whether this is a causative association, what underlying mechanism of unfavourable clinical outcomes in patients with low *MTAP* expression, and whether *MTAP* low expression may lead to enhanced sensitivity to PRMT5 inhibitors remain to be studied.

## AUTHOR CONTRIBUTIONS

Yiyu Xiao and Qianqian Peng: data analysis, validation and visualization, writing‐original draft, reviewing‐editing and methodology. Advaith Maya Sanjeev Kumar: data curation, analyses and validation, methodology. Houda Alachkar: conceptualization, resources, supervision, funding, validation, writing original draft, project administration, writing review and editing. All authors contributed to the article and approved the submitted version.

## CONFLICT OF INTEREST STATEMENT

The authors declare no conflict of interest.

## FUNDING INFORMATION

This study was supported by the University of Southern California Grant no. NIH‐NCI 1R01CA248381‐01A1 and National Institutes of Health (NIH) Grant no. 5P30CA014089‐45.

## ETHICS STATEMENT

Ethical approval was not required for the studies involving humans because this study was conducted on a publicly available dataset. The studies were conducted in accordance with the local legislation and institutional requirements. Written informed consent to participate in this study was not required from the participants or the participants’ legal guardians/next of kin in accordance with the national legislation and the institutional requirements.

## Supporting information

Supporting Information

## Data Availability

All the data used in this paper are publicly available.
