# Supplementary material for: Low methylthioadenosine phosphorylase expression is associated with worse survival in patients with acute myeloid leukaemia
Source: Clin Transl Med. 2024 Sep 11;14(9):e70015. doi: 10.1002/ctm2.70015 (PMC11389529; doi:10.1002/ctm2.70015)
Supplement: Supplementary file 1 — Supporting Information [file CTM2-14-e70015-s001.docx]

**Supplementary material:**

**Low MTAP expression is associated with worse survival in patients with Acute Myeloid Leukemia**

**Authors: Yiyu Xiao^1,^*, Qianqian Peng^1,^*, Advaith Maya Sanjeev Kumar^2,3^, Houda Alachkar ^3,4^**

**Affiliations:**

1. Department of Pharmacology and Pharmaceutical Sciences, USC Alfred E. Mann School of Pharmacy and Pharmaceutical Sciences, University of Southern California

2. Department of Computer Science, University of Southern California, Los Angeles, CA, United States.

3. Department of Clinical Pharmacy, USC Alfred E. Mann School of Pharmacy and Pharmaceutical Sciences, University of Southern California

4. USC Norris Comprehensive Cancer Center, University of Southern California

**Corresponding Author:**

Houda Alachkar, PharmD, PhD

Associate Professor

USC Alfred E. Mann School of Pharmacy and Pharmaceutical Sciences

University of Southern California

1985 Zonal Avenue, PSC 608

Los Angeles, CA 90089

Phone: 323-442-2696

Email: alachkar@usc.edu

*Contributed equally to the work

***Keywords***: Methylthioadenosine phosphorylase; Acute myeloid leukemia; 5’-deoxy-5'-methylthioadenosine; Protein Arginine Methyltransferase 5

**Supplementary Methods:**

**Patients’ data**

Microarray-based *MTAP* expression profiling of MILE (Microarray Innovations in Leukemia) study was downloaded from Blood Spot^1,2^, by querying ‘*MTAP*’. This study included 3,334 patients and 2096 samples, with 542 AML samples and 73 non-leukemia and healthy bone marrow were identified.

TCGA TARGET GTEx database in UCSC Xena^3^ was utilized to generate the comparison of *MTAP* gene and *MTAP* transcripts expression between normal peripheral blood samples from the Genotype-Tissue Expression (GTEx) project and AML samples from the Cancer Genome Atlas (TCGA) datasets^4,5^. Transcripts of *MTAP* included in UCSC Xena referenced the Release 23 (GRCh38.p3) of GENECODE reference annotation^6^.

TCGA AML dataset (NEJM 2013)^4^ and Oregon Health and Science University (OHSU) AML dataset (Cancer Cell 2022)^7^ were downloaded from cBioPortal v5.4.7^8–10^. TCGA dataset includes 200 samples of primary AML obtained at diagnosis from adult cases and 200 patients, with RNA-seq data obtained from 173 patients (median age: 58 years old; range: 18-88 years old)^4^. OHSU dataset had a cohort of 805 adult patients with AML and 942 samples^7^.

***MTAP* gene expression analysis**

TCGA dataset included 200 patients, 173 of the patients have RNAseq data for which mRNA expression z-scores relative to all samples (log RNA Seq V2 RSEM) were obtained. OHSU dataset included 805 patients and 942 samples, with 905 AML samples with RNA seq data (log RNA Seq RPKM). According to z-score relative to all samples, patients and samples were dichotomized into *MTAP* expression ‘*MTAP* low’ (z-score < -1) or ‘*MTAP* unaltered/high’ (z-score >-1).

**Statistical analyses**

For the comparison between *MTAP* expression in healthy bone marrow and AML specimens, Mann–Whitney U test and the Welch's t-test were applied to assess differences in the medians or means respectively between groups. Log-rank (Mantel-Cox) test was used to compare the survival of patients with low or unaltered/high *MTAP* expression. Cox proportional hazards regression models for multivariable analysis were developed. The model included parameters that were found significant in the univariable analysis based on P < 0.1. Fisher's exact test was used to assess the association between *MTAP* expression level and the frequencies of AML common mutations. The Mann-Whitney test and unpaired t test were used to compare *MTAP* expression levels according to the mutational status of patients. *MTAP* expression levels were also compared between patients within subgroups according to the French American (FAB) classifications provided in the dataset. The Mann-Whitney test and the Wilcoxon test were applied to compare *MTAP* expression according to disease status (diagnosis, relapse, remission, residual disease) between unpaired and paired samples, respectively. The Dunn’s multiple comparisons test and Bonferroni correction test were used to adjust p values for multiple testing. All figures were generated by GraphPad Prism (Version 10.1.0).

**Supplementary Tables:**

Table S1. Clinicopathological features (TCGA)

| **Clinicopathological features** |  | **n** | **MTAP expression, n (%)** | | | ***P*** |
| --- | --- | --- | --- | --- | --- | --- |
|  |  |  | **Low**  **(n=18)** | **Unaltered/high**  **(n=155)** |  | |
| **Diagnosis Age** |  |  |  |  | | **0.0276*** |
|  | <65y | 120 | 8 (6.67) | 112 (93.33) | |  |
|  | ≥65y | 53 | 10 (18.87) | 43 (81.13) | |  |
| **Sex** |  |  |  |  | | 0.2211 |
|  | Male | 92 | 7 (7.61) | 85 (92.39) | |  |
|  | Female | 81 | 11 (13.58) | 70 (86.42) | |  |
| **Bone marrow blast percentage**  **(Median: 72)** |  |  |  |  | | 0.6191 |
|  | <72 | 82 | 10 (12.20) | 72 (87.80) | |  |
|  | ≥72 | 91 | 8 (8.79) | 83 (91.21) | |  |
| **White blood cell count**  **(Median: 17)** |  |  |  |  | | 0.1432 |
|  | <17 | 86 | 12 (13.95) | 74 (86.05) | |  |
|  | ≥17 | 87 | 6 (6.90) | 81 (93.10) | |  |
| **Peripheral blasts percentage**  **(Median: 39)** |  |  |  |  | | 0.6224 |
|  | Low | 83 | 10 (12.05) | 73 (87.95) | |  |
|  | High | 87 | 8 (9.20) | 79 (90.80) | |  |
|  | N/A** | 3 | 0 (0.00) | 3 (100.00) | |  |
| **Cytogenetic Risk***** |  |  |  |  | | 0.5967 |
|  | Good | 32 | 2 (6.25) | 30 (93.75) | |  |
|  | Intermediate | 101 | 12 (11.88) | 89 (88.12) | |  |
|  | Poor | 37 | 4 (10.81) | 33 (89.19) | |  |
|  | N/A** | 3 | 0 (0.00) | 3 (100.00) | |  |
| **Molecular risk***** |  |  |  |  | | 0.5646 |
|  | Good | 33 | 2 (6.06) | 31 (93.94) | |  |
|  | Intermediate | 92 | 12 (13.04) | 80 (86.96) | |  |
|  | Poor | 45 | 4 (8.89) | 41 (90.11) | |  |
|  | N/A | 3 | 0 (0.0) | 3 (100.0) | |  |

*P < 0.05, **P < 0.01, ***P < 0.001, and ns means no significance found.

Table S2. Clinicopathological features (OHSU)

| **Clinicopathological features** |  | **n** | **MTAP expression, n (%)** | | ***P*** | |
| --- | --- | --- | --- | --- | --- | --- |
|  |  |  | **Low**  **(n=52)** | **Unaltered/high**  **(n=386)** | |  |
| **Diagnosis Age** |  |  |  |  | 0.2870 | |
|  | <65y | 263 | 27 (10.27) | 236 (89.73) |  | |
|  | ≥65y | 173 | 24 (13.87) | 149 (86.13) |  | |
| **Sex** | N/A | 2 | 1 | 1 |  | |
|  |  |  |  |  | 0.0537 | |
|  | Male | 238 | 35 (8.50) | 203 (91.50) |  | |
|  | Female | 200 | 17 (14.71) | 183 (85.29) |  | |

Table S3. Mutations in patients (TCGA)

| **Mutations** |  | **n** | **MTAP expression, n (%)** | | ***P*** | | ***Adjusted P*** |
| --- | --- | --- | --- | --- | --- | --- | --- |
|  |  |  | **Low**  **(n=18)** | **Unaltered/high**  **(n=155)** | |  |  |
|  |  |  |  |  |  | |  |
| **FLT3** |  |  |  |  | 0.783 | |  |
|  | FLT3-wt | 124 | 14 (77.8) | 110 (71.0) |  | |  |
|  | FLT3-mut | 49 | 4 (22.2) | 45 (29.0) |  | |  |
| **NPM1** |  |  |  |  | 0.782 | |  |
|  | NPM1-wt | 125 | 14 (77.8) | 111 (71.6) |  | |  |
|  | NPM1-mut | 48 | 4 (22.2) | 44 (28.4) |  | |  |
| **DNMT3A** |  |  |  |  | 1.000 | |  |
|  | DNMT3A-wt | 131 | 14 (77.8) | 117 (75.5) |  | |  |
|  | DNMT3A-mut | 42 | 4 (22.2) | 38 (24.5) |  | |  |
| **IDH2** |  |  |  |  | 0.082 | |  |
|  | IDH2-wt | 156 | 14 (77.8) | 142 (91.6) |  | |  |
|  | IDH2-mut | 17 | 4 (22.2) | 13 (8.4 |  | |  |
| **IDH1** |  |  |  |  | 1.000 | |  |
|  | IDH1-wt | 157 | 17 (94.4) | 140 (90.3) |  | |  |
|  | IDH1-mut | 16 | 1 (5.6) | 15 (9.7) |  | |  |
| **TP53** |  |  |  |  | 1.000 | |  |
|  | TP53-wt | 159 | 17 (94.4) | 142 (91.6) |  | |  |
|  | TP53-mut | 14 | 1 (5.6) | 13 (8.4) |  | |  |

* *P* values were from Fisher’s Exact test

** Adjusted *P* values were from Bonferroni adjustment

Table S4. Mutations in patients (OHSU)

| **Mutation** |  | **n** | **MTAP expression, n (%)** | | ***P*** | | ***Adjusted P*** |
| --- | --- | --- | --- | --- | --- | --- | --- |
|  |  |  | **Low**  **(n=52)** | **Unaltered/high**  **(n=389)** | |  |  |
| **FLT3** |  |  |  |  | **0.036*** | | 0.216 |
|  | FLT3-wt | 307 | 43 (82.7) | 264 (67.9) |  | |  |
|  | FLT3-mut | 134 | 9 (17.3) | 125 (32.1) |  | |  |
| **NPM1** |  |  |  |  | **0.000***** | | **0.000***** |
|  | NPM1-wt | 320 | 50 (96.2) | 270 (69.4) |  | |  |
|  | NPM1-mut | 121 | 2 (3.8) | 119 (30.6) |  | |  |
| **DNMT3A** |  |  |  |  | 0.575 | |  |
|  | DNMT3A-wt | 356 | 44 (84.6) | 312 (80.2) |  | |  |
|  | DNMT3A-mut | 85 | 8 (15.4) | 77 (19.8) |  | |  |
| **IDH2** |  |  |  |  | 0.818 | |  |
|  | IDH2-wt | 392 | 46 (88.5) | 346 (88.9) |  | |  |
|  | IDH2-mut | 49 | 6 (11.5) | 43 (11.1) |  | |  |
| **IDH1** |  |  |  |  | 0.769 | |  |
|  | IDH1-wt | 411 | 48 (92.3) | 363 (93.3) |  | |  |
|  | IDH1-mut | 30 | 4 (7.7) | 26 (6.7) |  | |  |
| **TP53** |  |  |  |  | 0.579 | |  |
|  | TP53-wt | 407 | 47 (90.4) | 360 (92.5) |  | |  |
|  | TP53-mut | 34 | 5 (9.6) | 29 (7.5) |  | |  |

References

1. Haferlach T, Kohlmann A, Basso G, et al. The Clinical Utility of Microarray-Based Gene Expression Profiling in the Diagnosis and Sub-Classification of Leukemia: Final Report on 3252 Cases from the International MILE Study Group. *Blood*. 2008;112(11):753. doi:10.1182/blood.V112.11.753.753

2. Bagger FO, Sasivarevic D, Sohi SH, et al. BloodSpot: a database of gene expression profiles and transcriptional programs for healthy and malignant haematopoiesis. *Nucleic Acids Research*. 2016;44(D1):D917-D924. doi:10.1093/nar/gkv1101

3. Goldman MJ, Craft B, Hastie M, et al. Visualizing and interpreting cancer genomics data via the Xena platform. *Nat Biotechnol*. 2020;38(6):675-678. doi:10.1038/s41587-020-0546-8

4. Genomic and Epigenomic Landscapes of Adult De Novo Acute Myeloid Leukemia. *N Engl J Med*. 2013;368(22):2059-2074. doi:10.1056/NEJMoa1301689

5. Lonsdale J, Thomas J, Salvatore M, et al. The Genotype-Tissue Expression (GTEx) project. *Nat Genet*. 2013;45(6):580-585. doi:10.1038/ng.2653

6. Frankish A, Diekhans M, Ferreira AM, et al. GENCODE reference annotation for the human and mouse genomes. *Nucleic Acids Research*. 2019;47(D1):D766-D773. doi:10.1093/nar/gky955

7. Bottomly D, Long N, Schultz AR, et al. Integrative analysis of drug response and clinical outcome in acute myeloid leukemia. *Cancer Cell*. 2022;40(8):850-864.e9. doi:10.1016/j.ccell.2022.07.002

8. de Bruijn I, Kundra R, Mastrogiacomo B, et al. Analysis and Visualization of Longitudinal Genomic and Clinical Data from the AACR Project GENIE Biopharma Collaborative in cBioPortal. *Cancer Research*. Published online September 5, 2023. doi:10.1158/0008-5472.CAN-23-0816

9. Cerami1 E, Gao J, Dogrusoz U, et al. The cBio Cancer Genomics Portal: An Open Platform for Exploring Multidimensional Cancer Genomics Data. *Cancer Discov*. 2012;2(5):401-404. doi:10.1158/2159-8290.CD-12-0095

10. Gao J, Aksoy BA, Dogrusoz U, et al. Integrative analysis of complex cancer genomics and clinical profiles using the cBioPortal. *Sci Signal*. 2013;6(269):pl1. doi:10.1126/scisignal.2004088
